# Identification of novel subtypes based on ssGSEA in immune‐related prognostic signature for tongue squamous cell carcinoma

**DOI:** 10.1002/cam4.4341

**Published:** 2021-10-20

**Authors:** Yi Jin, Zhanwang Wang, Dong He, Yuxing Zhu, Xingyu Chen, Ke Cao

**Affiliations:** ^1^ Department of Oncology Third Xiangya Hospital of Central South University Changsha China; ^2^ Department of Radiation Oncology Hunan Cancer Hospital, The Affiliated Cancer Hospital of Xiangya School of Medicine Central South University Changsha China; ^3^ Key Laboratory of Translational Radiation Oncology Department of Radiation Oncology Hunan Cancer Hospital and The Affiliated Cancer Hospital of Xiangya School of Medicine Central South University Changsha China; ^4^ Department of Respiratory The Second People’s Hospital of Hunan Province Changsha China

**Keywords:** CIBERSORT, ESTIMATE, LASSO, PGK1, ssGSEA, tongue squamous cell carcinoma

## Abstract

**Background:**

Tongue squamous cell carcinoma (TSCC) is characterized by aggressive invasion and poor prognosis. Currently, immune checkpoint inhibitors may prolong overall survival compared with conventional treatments. However, PD1/PDL1 remain inapplicable in predicting the prognosis of TSCC; thus, it is urgent to explore the genetic characteristics of TSCC.

**Materials and methods:**

We utilized single‐sample gene set enrichment analysis (ssGSEA) to classify TSCC patients from the TCGA database into clusters with different immune cell infiltrations. ESTIMATE (immune‐related scores) and CIBERSORT (immune cell distribution) analyses were used to evaluate the immune landscape among clusters. GO, KEGG, and GSEA analyses were performed to analyze the different underlying molecular mechanisms in the clusters. Based on the immune characteristics, we applied the LASSO Cox regression to select hub genes and construct a prognostic risk model. Finally, we established an interactive network among these hub genes by using Cytoscape, and a pan‐cancer analysis to further verify and decipher the innate function of these genes.

**Results:**

Using ssGSEA, we constructed three functional clusters with different overall survival and immune‐cell infiltration. ESTIMATE and CIBERSORT analyses revealed the different distributions of immune cells (T cells, B cells, and macrophages) with diverse immune‐related scores (ESTIMATE, immune, stromal, and tumor purity scores). Moreover, pathways including those of the interferon‐gamma response, hypoxia, and glycolysis of the different subtypes were investigated to elucidate their involvement in mediating the heterogeneous immune characteristics. Subsequently, after LASSO Cox regression, a signature of 15 immune‐related genes was established that is more prognostically effective than the TNM stage. Furthermore, three hub genes—*PGK1*, *GPI*, and *RPE*—were selected using Cytoscape evaluation and verified by immunohistochemistry. *PGK1*, the foremost regulator, was a comprehensively profiled pan‐cancer, and a *PGK1*‐based interactive network was established.

**Conclusion:**

Our results suggest that immune‐related genes and clusters in TSCC have the potential to guide individualized treatments.

## Graphical abstract

Our findings suggested that these immune‐related genes may act as promising indicators for mediating the immune microenvironment characterizations and providing novel insight into immunotherapy.

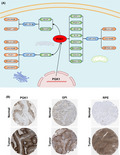

## 1 INTRODUCTION

Head and neck squamous cell carcinoma (HNSCC) is the sixth most common malignancy globally, and its incidence has been significantly increasing worldwide.^1^ HNSCC arises from the squamous epithelium of the oral cavity, oropharynx, larynx, and hypopharynx and represents a heterogeneous group of tumors. Tongue squamous cell carcinoma (TSCC) is one of the most frequent types of cancer, particularly of the oral cavity, and is characterized by aggressive local invasion and early lymph‐node metastasis with a relatively poor prognosis.^2^, ^3^ Environmental carcinogens such as tobacco, alcohol, or HPV infection may induce the development of TSCC.^4^ Conventional treatments for TSCC, including surgical techniques, chemoradiotherapy, and some other target therapies, result in inevitable short‐term and long‐term morbidity; it has been difficult to improve the overall survival (OS) of patients with TSCC, which has been approximately 50% over recent decades.^5^ The tumor, lymph node, and metastasis (TNM) classification system has been widely used and is considered a vital tool in predicting the survival outcomes of TSCC patients. However, this system has been criticized because it cannot be used to decipher the heterogeneous outcomes of individual cancers with the same TNM stage. Thus, it is crucial to reveal the biologic molecular mechanism underlying TSCC development and to discover novel biomarkers for the prediction of clinical outcomes.

Recent research has led to the discovery of several novel biomarkers linked to the establishment and progression of TSCC, especially those involving the tumor genes *TP53* ^6^ and *EGFR*.^7^ Moreover, cetuximab, a monoclonal antibody that targets EGFR, has been shown to have efficacy in locoregionally advanced, recurrent, or metastatic HNSCC in combination with chemotherapy or radiation.^8^ However, the efficacy of such treatment with curative intent is limited to the best median OS of about 10 months, and this treatment is associated with substantial toxicity.^9^ Furthermore, there are no approved treatment options after TSCC progression.^10^ According to the KEYNOTE‐048, a randomized, open‐label, phase III study, the results suggest that pembrolizumab monotherapy, which targets the programmed cell death 1 (PD‐1) receptor,^11^ has significantly longer OS and a favorable safety profile in the population with PD‐L1 combined positive score (CPS) ≥20 compared with cetuximab plus chemotherapy.^9^ Nowadays, the field of immunology has attracted much attention for its irreplaceable clinical benefits. Innate immunologic mechanisms are often observed to correlate with the mediation of the tumor microenvironment, which is composed of immune cells and stromal cells, among others.^12^ There is no denying that immune‐related genes and immune infiltrating cells have indispensable roles in the tumor microenvironment; yet, little is known regarding the immune landscape of TSCC.^13^ Therefore, it is crucial to comprehensively analyze immune‐related cells or genes in TSCC to further decipher the mechanism underlying immunology resistance and response.

In this study, we used single‐sample gene set enrichment analysis (ssGSEA) to assign TSCC patients from the Cancer Genome Atlas (TCGA) database into different immune‐cell infiltration clusters; we used ESTIMATE, CIBERSORT, and K‐M analyses to identify the prognostic significance of immune‐related risk models or regulators and explored the mechanisms underlying the characterization of the tumor microenvironment.

## 2 MATERIALS AND METHODS

### 2.1 Data processing

The RNA‐seq data (FPKM value) and clinical characteristics (including age, sex, smoking status, tumor stage, and TNM staging) of the TSCC cohort were obtained from TCGA Head and Neck Squamous Cell Carcinomas database. We included samples wherein the primary site was the tongue and obtained publicly available data.^14^

### 2.2 ssGSEA and hierarchical clustering analysis

The ssGSEA algorithm was based on 29 immune gene sets, including genes related to different immune cell types, functions, pathways, and checkpoints. We employed the ssGSEA algorithm via R packages (GSVA, GSEABase, and limma) to comprehensively assess the immunologic characteristics of every sample included in the study.^15^

To study the correlation between immunity and clinical phenotype of TSCC, we used “Consensus Cluster Plus” (50 iterations, 80% resampling rate) according to the enrichment score of immune items in ssGSEA to cluster the TSCC samples into three different groups (Clusters A, B, and C). OS analysis between different clusters was conducted using the Kaplan–Meier method.

### 2.3 ESTIMATE and CIBERSORT analyses

To verify differences in the immunity of the three clusters, we utilized the “estimate” package for R (https://sourceforge.net/projects/estimateproject) to calculate the tumor purity, stromal score, immune score, and ESTIMATE score of each TSCC tumor sample.^16^ The Mann–Whitney *U* test was used to compare the scores of the three clusters. Then, we used the CIBERSORT package to assess the distribution of 22 immune cell types in each sample. Results with *p* < 0.05 were used in further analysis.^17^

### 2.4 Gene Ontology (GO) analysis, Kyoto Encyclopedia of Genes and Genomes (KEGG) database analysis, and GSEA

The “edgeR” package in R was used to perform a differential analysis of the mRNAs in the low‐expression and high‐expression groups.^18^ Differentially expressed genes (DEGs) were selected based on the following significance criteria: |logFC| >1 and FDR <0.05. We further performed GO functional annotation, including molecular function, cellular component, and biologic process analysis. The KEGG database analyzes the metabolic pathways and signal transduction pathways in which DEGs are significantly enriched. GSEA was then performed to identify the signaling pathways wherein DEGs were enriched between the high‐risk and low‐risk subgroups.^19^

### 2.5 Construction of the prognostic risk model

The “survival” package in R was used to perform univariate Cox proportional hazard regression analysis to screen for immune‐related genes that are significantly linked to the OS of TSCC patients in the TCGA cohort. The “Glmnet” package was used to perform LASSO Cox regression analysis.^20^ We calculated the risk score to construct the risk model using the following formula: risk score =expressed mRNA_1_ × coefficient mRNA_1_ + expressed mRNA_2_ × coefficient mRNA_2_ + …expressed mRNA*_n_* × coefficient mRNA*_n_*. Patients were divided into high‐risk and low‐risk groups according to the value of the risk coefficient. The Kaplan‐Meier survival package in R language was used to perform univariate and multivariate Cox proportional hazard regression analyses on the risk value. The Cox analysis signature included gender, age, grade, and risk score, as well as T, N, and M stages. The sensitivity and specificity of the receiver‐operating characteristic (ROC) curve were used to evaluate the prognostic performance of the signature.

### 2.6 Identification and verification of immune‐related hub genes

To understand the underlying interaction of immune‐related mRNAs, the STRING website (https://string‐db.org/) was employed, and all PPI pairs with a combined score of >0.4 were extracted. Next, we utilized the Cytoscape (v3.6.1) plugin cytoHubba to calculate all degrees of nodes.^21^ In the present study, genes with the highest degree values were considered hub genes. The Human Protein Atlas (HPA) online database (http://www.proteinatlas.org/) was used to validate the expression of hub genes at the translational level.

### 2.7 Identification of the interaction of immune‐related hub genes

To further explore the interaction of hub genes with miRNAs, lncRNAs, and circRNAs, with the criteria of |log2(fold change)| >1 and *p* < 0.05, we identified differentially expressed lncRNAs and miRNAs in the TCGA–TSCC cohort. Furthermore, we used the miRcode database (mircode.org/index.php) to target miRNA, and we utilized miRTarBase (http://mirtarbase.mbc.nctu.edu.tw/) and miRDB (http://www.mirdb.org/) to identify the correlation between miRNA and mRNA. Subsequently, we screened circRNA–miRNA interaction pairs using the circBank database (http://www.circbank.cn/index.html). Finally, after integrating circRNA–miRNA, lncRNA–miRNA, and miRNA–mRNA regulatory relationships, a competing endogenous RNA (ceRNA) network was established and visualized.

### 2.8 Statistical analysis

All analyses were performed using R. Univariate and multivariate Cox proportional hazards regression analyses were also used to assess the relationship between the risk score and OS of patients. ROC analysis was used to detect the sensitivity and specificity of the genetic signature risk score to predict survival. The area under the ROC curve (AUC) was used as an index to assess the accuracy of the prognosis. In all analyses, a *p* value of <0.05 was considered to be statistically significant.

## 3 RESULTS

### 3.1 Construction of TSCC groupings based on ssGSEA

**FIGURE 1:**
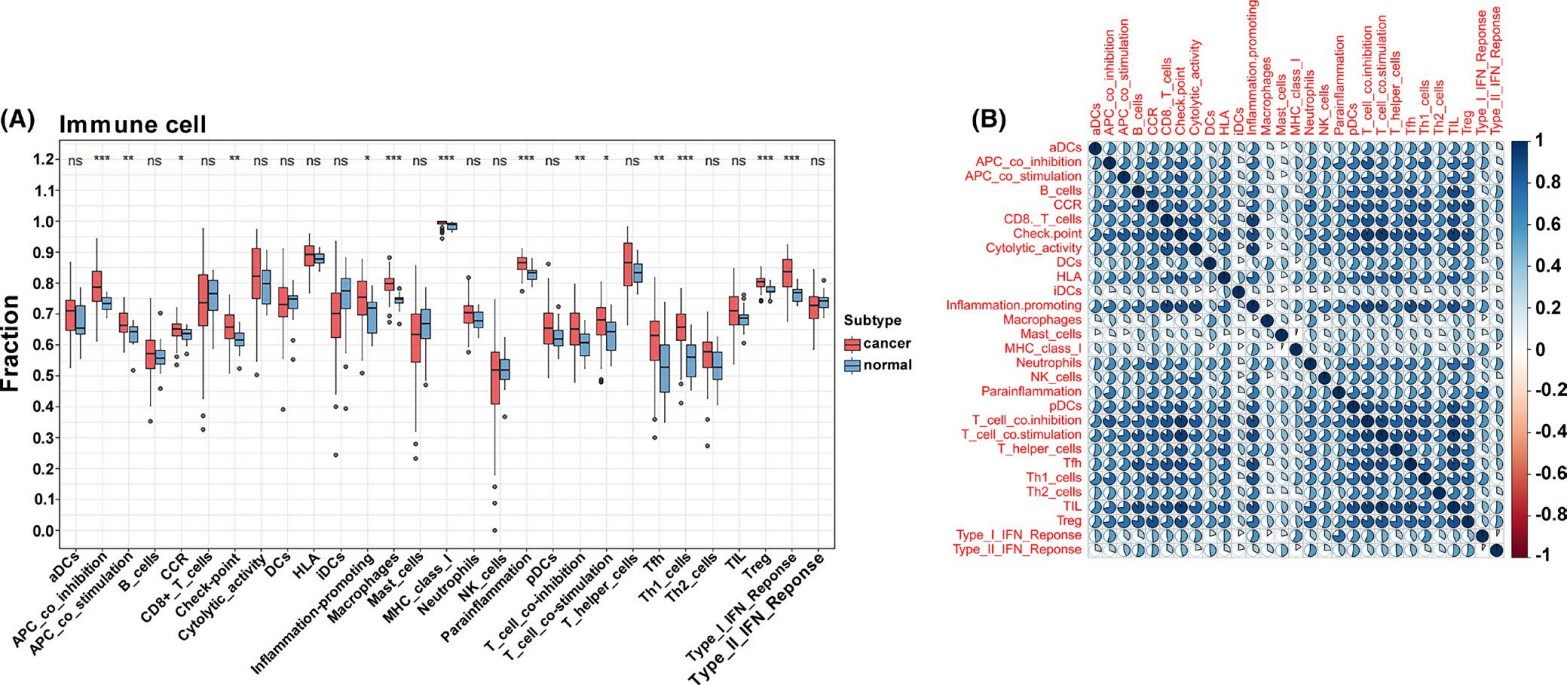
The identification of immune gene sets by ssGSEA. (A) The expression of immune gene sets by ssGSEA in cancer and normal tissue. (B) The Pearson correlation of immune cell types in cancer tissue based on ssGSEA

In total, 147 TSCC samples and 15 paracancerous samples were obtained from the TCGA database. The ssGSEA method was applied to the transcriptome of the TSCC samples to evaluate the distribution of 29 immune cell types. As shown in Figure 1A, over half of the immune cell types were found to be upregulated in the TSCC tissue than in normal tissue. The Pearson correlation analysis showed that these immune cell types in the cancer tissue intimately reinforced each other (Figure 1B), suggesting that TSCC is heterogeneous cancer with high immunogenicity.

**FIGURE 2:**
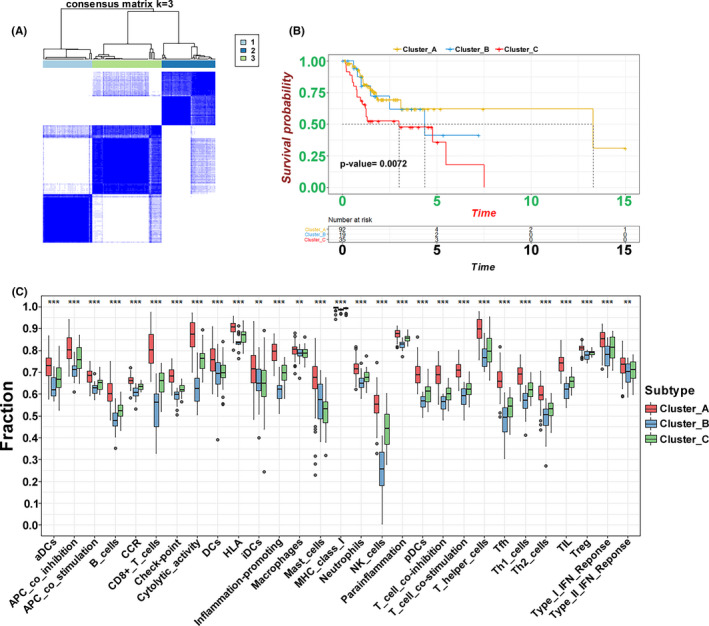
The classification of clusters identified by ssGSEA. (A) The Consensus Cluster package‐based clustering of patients identified by ssGSEA with *k* = 3. (B) The Kaplan–Meier curves of three clusters regarding OS. (C) The different expression of immune cell sets in the three clusters (***p < 0.001, **p < 0.01, *p < 0.05)

**FIGURE 3:**
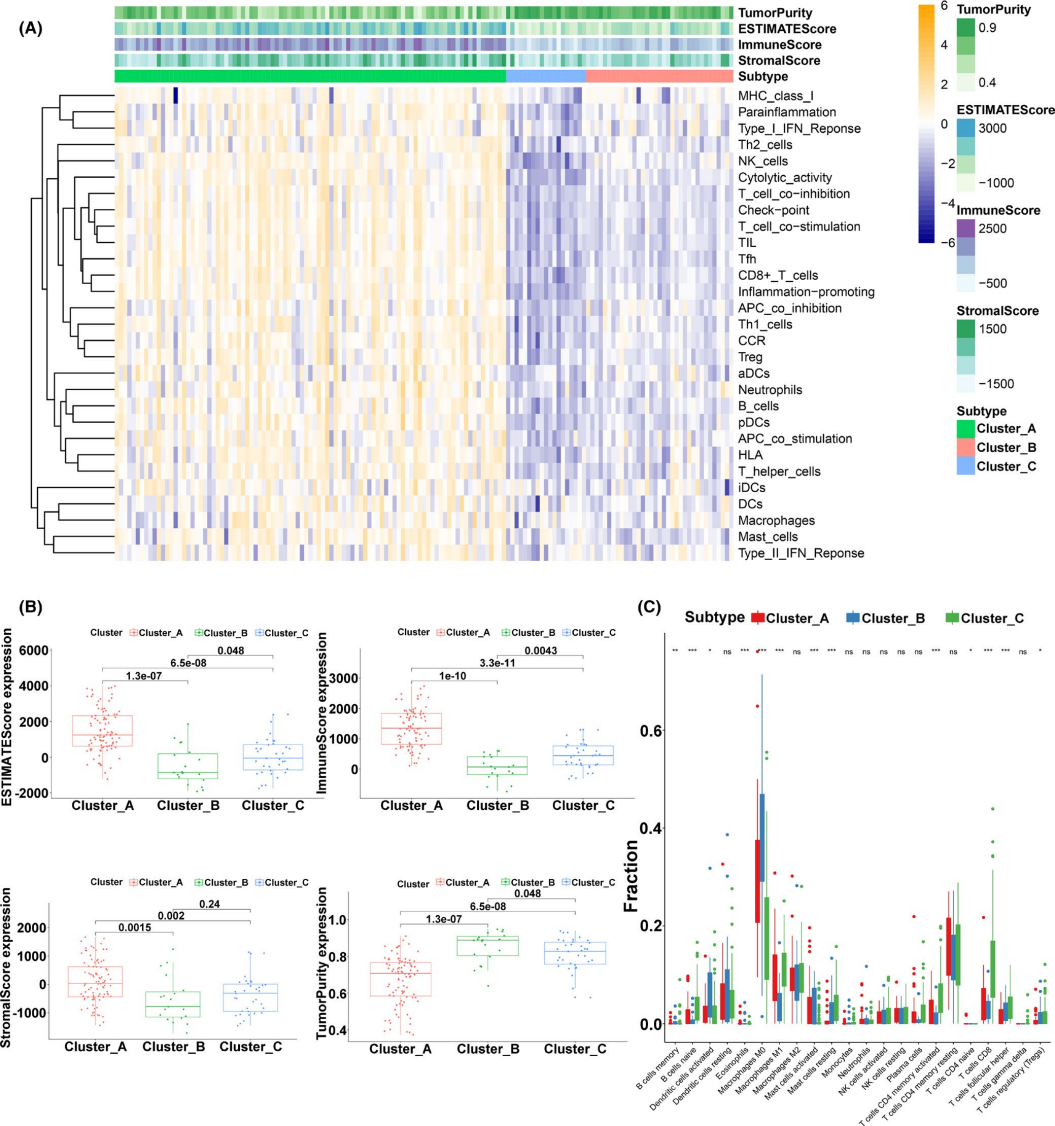
Immune landscape of three clusters. (A) The heatmap of immune gene sets identified by ssGSEA and ESTIMATE scores for the three clusters. (B) Different ESTIMATE scores, immune scores, stromal scores, and tumor purity in three clusters. (C) Different distributions of tumor‐infiltrating cells in three clusters (***p < 0.001, **p < 0.01, *p < 0.05)

To further understand the correlations among these immune cell types, we implemented a consensus clustering analysis to classify the TSCC samples into three clusters based on the ssGSEA using the Consensus Cluster Plus package with *k* = 3 (Figure 2A). The Kaplan–Meier method showed that there was a significant difference among clusters, particularly between clusters A and C (Figure 2B). Subsequently, we plotted a boxplot and heatmap to screen the relationship between immune‐related groupings and expression of gene sets from ssGSEA. The expression of most immune cell types was higher in cluster A with the low OS than in clusters B and C (Figures 2C and 3A).

### 3.2 Immune characterization in the TSCC clusters

To verify the feasibility of the grouping strategy, we conducted an ESTIMATE analysis to profile the immune characteristics of TSCC based on the expression of immune cell types. Stromal, immune, and ESTIMATE scores had a similar trend as the expression of immune cell types, which were upregulated in the relatedly high immune‐cell infiltration group (cluster A) (Figure 3A). The box chart also further verified that there the high immune‐cell infiltration cluster was significantly positively correlated with the ESTIMATE and immune and stromal scores and negatively correlated with tumor purity (Figure 3B). In addition, we used the CIBERSORT method to quantify the level of immune cell infiltration to carefully evaluate the immune landscape. The results showed that the types of B cells, T cells, and macrophages contributed to immune cell infiltration. We observed that the levels of naïve B cells, CD4 memory‐activated T cells, CD8 T cells, and follicular helper T cells were remarkably increased in cluster C (Figure 3C). These results indicate that this ssGSEA‐based clustering strategy can be used to decipher the heterogeneous immune landscape of TSCC, and specific multiple immune‐related modulators may have an integrated effect in the mediation of the tumor microenvironment.

### 3.3 Interaction of immune‐related subgroups with different survivals

**FIGURE 4:**
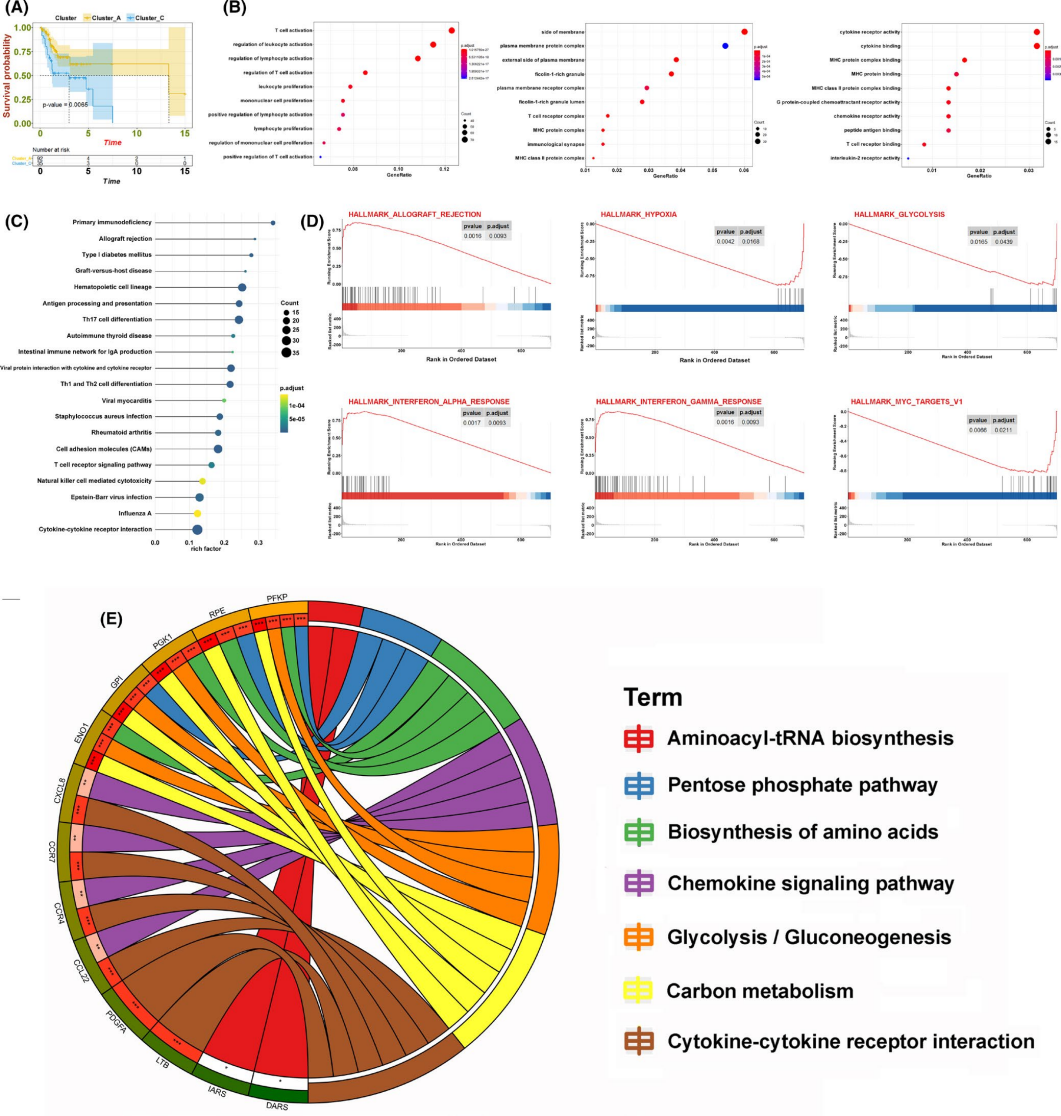
Relationship between cluster A and cluster C. (A) The OS Kaplan–Meier curves of cluster A and cluster C. (B) OS analysis of all DEGs from the two clusters. (C) KEGG analysis of all DEGs from the two clusters. (D) GSEA of all DEGs from the two clusters. (E) Circos plot of 41 prognostic mediators from the two clusters

To investigate the potential mechanisms involved in the heterogeneity of TSCC, due to the small sample size, we included the samples of two clusters (clusters A and C) in the next analysis. Kaplan–Meier analysis revealed that the OS was significantly different in the two clusters (*p* = 0.0065) (Figure 4A). Based on both clusters, we identified 626 DEGs with the criteria of |log2(fold change)| >2 and adj.*p* < 0.05 in the TCGA‐TSCC cohort. Compared with cluster C, cluster A included DEGs associated with T cell activation and regulation of leukocyte activation, according to the biologic‐process GO analysis. Cellular component analysis showed that these DEGs were linked to the side of the membrane and the plasma membrane protein complex, and the molecular function analysis showed that cytokine receptor activity and cytokine binding were crucial (Figure 4B). The KEGG analysis indicated that some immune‐related pathways, such as primary immunodeficiency, Th17 cell differentiation, and Th1/Th2 cell differentiation, were enriched in both clusters (Figure 4C). Using GSEA analysis, these signaling pathways, including interferon‐gamma response, hypoxia, and glycolysis, were viewed to be core biologic carcinogenic processes involved in the regulation of the immune microenvironment (Figure 4D). Additionally, we performed univariate Cox analysis to select prognostic regulators from these DEGs, and 41 mRNAs were identified (Table S1). Finally, we continuously iterated the enrichment analysis of these 41 mediators and found that multiple pathways, especially glycolysis, not only played important roles in the regulation of the immune landscape but also altered clinical survival in TSCC (Figure 4E).

### 3.4 Construction of the immune risk model

**FIGURE 5:**
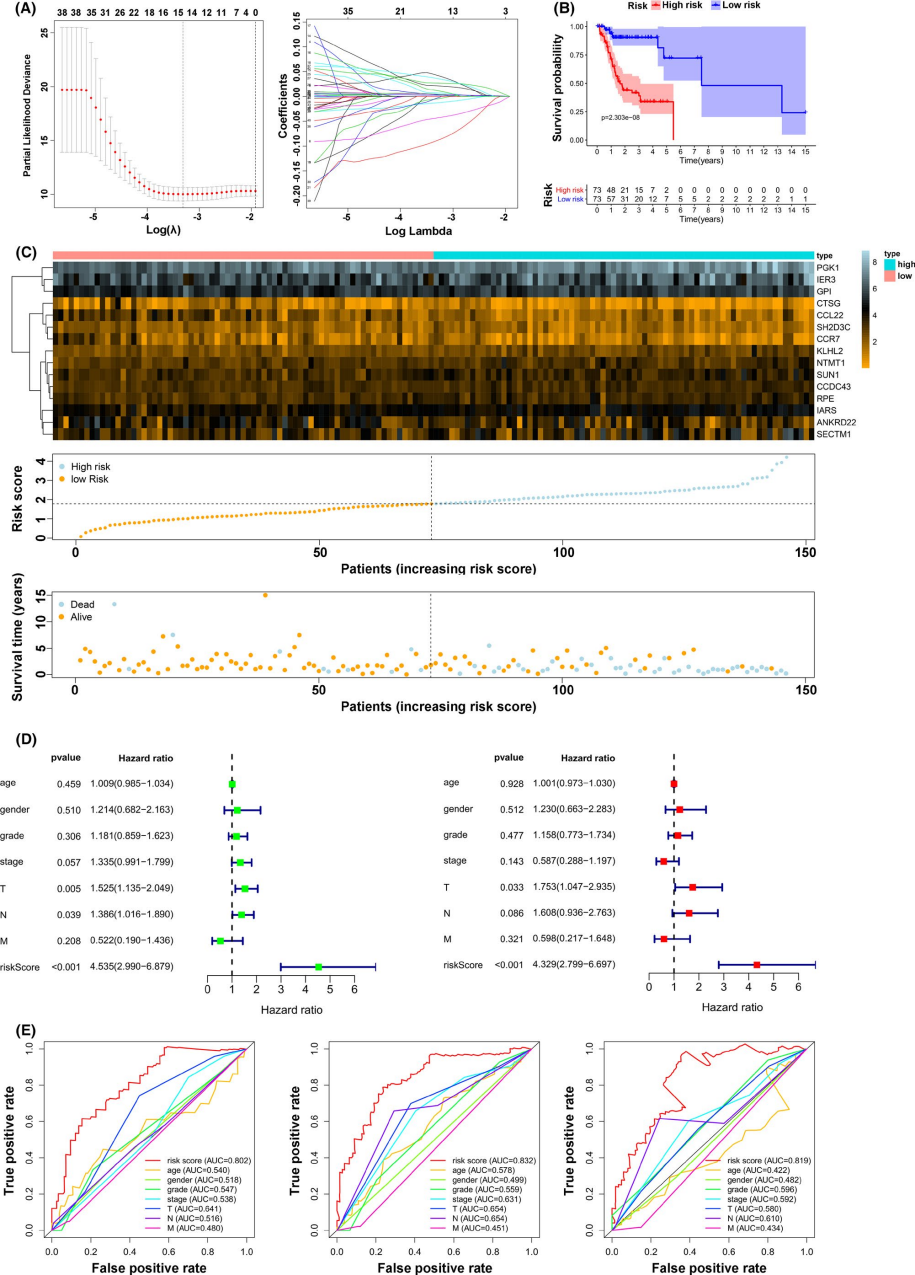
Establishment of the risk model. (A) Lasso Cox regression analysis of the 41 prognostic mediators. (B) The OS Kaplan–Meier analysis for patients in high‐/low‐risk groups. (C) Distributions of the 15 significant prognostic mediators, risk scores, and alive/dead status. (D) Univariate and multivariate analyses of age, gender, grade, and risk score and the T, N, and M stages. (E) The ROC curve of the risk model for 1‐year, 3‐year, and 5‐year survival

Based on the above findings, we applied Lasso Cox regression analysis to the 41 immune‐related mRNAs. Thereafter, we built a risk model with 15 mRNAs, and the coefficients of these genes were used to calculate the risk score as follows (Figure 5A): risk score = CCDC43 × 0.0034 + CCL22 × (−0.0436) + KLHL2 × 0.0429 + SH2D3C × (−0.0052) + ANKRD22 × (−0.0129) + SUN1 × 0.0215 + IARS × 0.0269 + NTMT1 × 0.0069 + IER3 × 0.0042 + CCR7 × (−0.089) + PGK1 × 0.0024 + CTSG × (−0.0038) + GPI × 0.0043 + SECTM1 × 0.0364 + RPE × 0.0119. Patients were divided into low‐risk and high‐risk groups according to the cutoff value (median risk score). The OS Kaplan‐Meier curve showed that the patients in the high‐risk group had worse OS than those in the low‐risk group (*p *= 2.303e^−8^) (Figure 5B). The risk coefficient and mortality of samples in the high‐risk group were higher than those in the low‐risk group (Figure 5C). Univariate and multivariate analyses showed that the risk score can be an independent prognostic biomarker. Unfortunately, after adjusting for all risk factors, only the T stage was slightly associated with OS (Figure 5D). To evaluate the prognostic effectiveness of the risk score, the ROC curve showed that the AUCs of 1‐year, 3‐year, and 5‐year survival were 0.802, 0.832, and 0.819, respectively, suggesting that the risk model was significantly effective and applicable (Figure 5E).

### 3.5 The immune landscape and verification of hub genes

**FIGURE 6:**
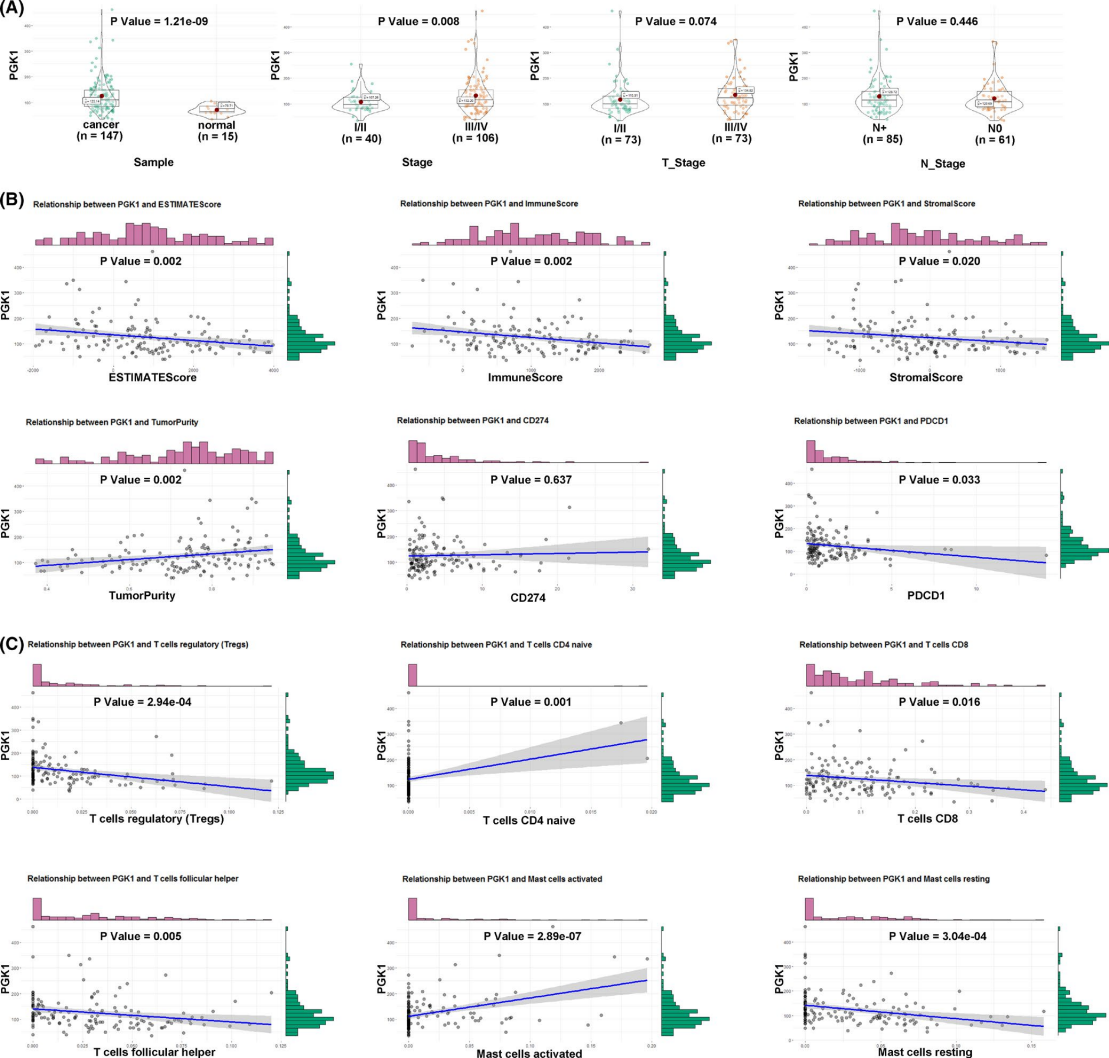
Comprehensive evaluation of *PGK1*. (A) The relationship between *PGK1* expression and clinical characteristics. (B) The relationship between *PGK1* expression and ESTIMATE scores, PD1 (PDCD1)/PD‐L1 (*CD274*). (C) The relationship between *PGK1* expression and tumor‐infiltrating cells

**FIGURE 7:**
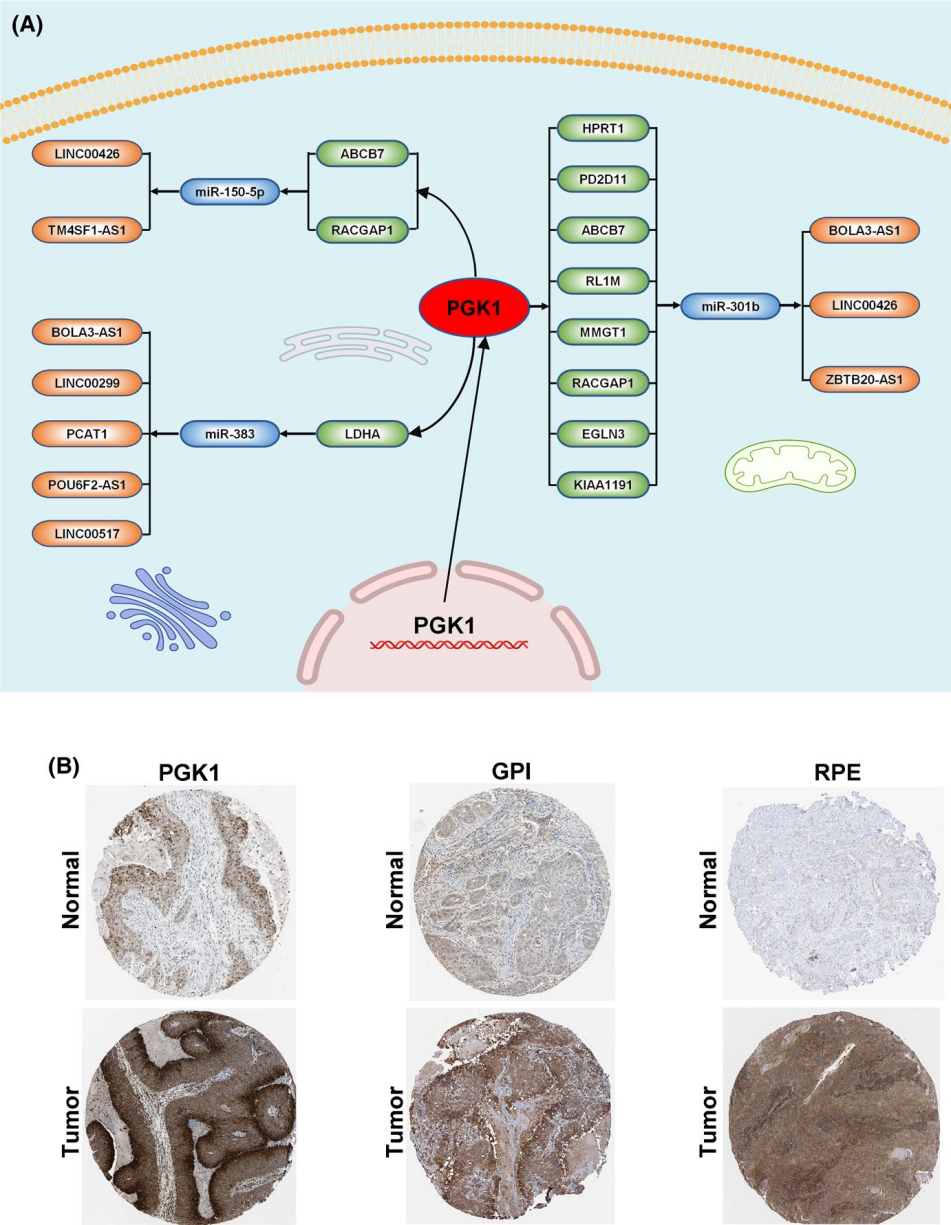
Interaction and verification of *PGK1* in TSCC. (A) The verification of *PGK1*, *GPI*, and *RPE* expression in immunohistochemistry. (B) The interactive network of *PGK1*

To precisely select the core regulators in the immune signature, we utilized Cytoscape based on the STRING website to calculate all degrees of nodes among the 15 regulators and identified the hub genes, *PGK1*, *GPI*, and *RPE*, which had the top three highest degree values (Table S2). To further confirm the key role of these hub genes in the immune mediation process, as shown in Figure 6A, *PGK1* expression was reevaluated and was found to be significantly highly correlated with clinical stage and tumorigenesis in TSCC. Moreover, the results revealed that the *PGK1* expression had an evident negative correlation with tumor purity (*p* = 0.002) and a positive correlation with the immune score (*p* = 0.002), stromal score (*p* = 0.020), and ESTIMATE score (*p* = 0.002). Additionally, we noted that the expression of *PGK1* was closely related to that of *PD1* (also known as *PDCD1* or *CD279*) (Figure 6B). Six types of immune cells that had an influence on TME, such as CD8 T cells and follicular helper T cells, were verified to be regulated by the hub gene *PGK1* (Figure 6C). The findings for *GPI* and *RPE* are presented in Figure S1. In accordance with the immunohistochemistry results from the Human Protein Atlas database, the expression of the hub genes *PGK1*, *GPI*, and *RPE* was obviously elevated in TSCC tissues than in normal tissues (Figure 7A).

### 3.6 Interaction and pan‐cancer analysis of *PGK1*

**FIGURE 8:**
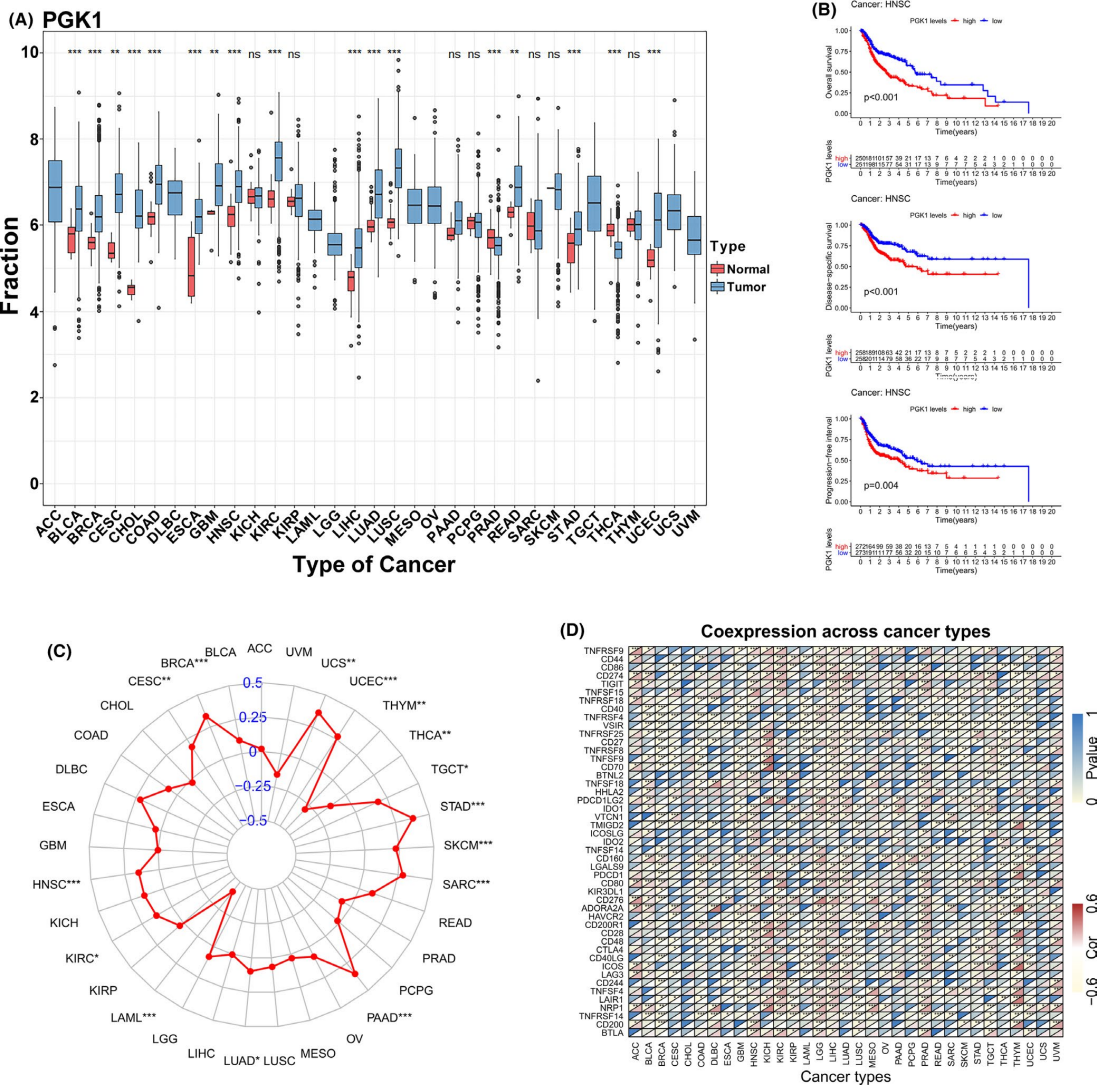
Pan‐cancer analysis of *PGK1*. (A) The expression of *PGK1* in 32 cancer species. (B) The relationship between *PGK1* expression and OS, DSS, and PFI in HNSCC. (C) The relationship between *PGK1* expression and TMB in 32 cancer species. (D) The relationship between *PGK1* expression and immune‐related regulators

To further unravel the interaction of *PGK1* in the immune‐related pathway with miRNAs, lncRNAs, and circRNAs, we first identified 13 lncRNAs and 18 miRNAs with |logFC(fold change)| ≥1 and FDR <0.05, and targeted 915 mRNAs (miRTarBase, miRDB). We identified the intersection of these 915 mRNAs and the top 100 *PGK1*‐related genes. Finally, we visualized and constructed a ceRNA regulatory network containing nine *PGK1*‐related mRNAs, three miRNAs, and 10 lncRNAs (Figure 7B). After determining the crucial molecular relationships of *PGK1*, we explored this oncogene in a pan‐cancer analysis. We downloaded all pan‐cancer data from the UCSC Cancer Genomics Browser (https://genome‐cancer.ucsc.edu). The results indicated that the expression of *PGK1* was distinctly upregulated in most types of cancers, including bladder urothelial carcinoma (BLCA), head and neck squamous cell carcinoma (HNSCC), and lung adenocarcinoma (LUAD) (Figure 8A). Furthermore, high *PGK1* expression was correlated with decreased OS (*p* < 0.001), disease‐specific survival (DSS) (*p* < 0.001), and progression‐free interval (PFI) (*p* = 0.004) (Figure 8B). Similarly, high *PGK1* expression was positively associated with tumor mutational burden (TMB), a promising indicator to differentiate responders to immune checkpoint inhibitors (Figure 8C). The pan‐cancer analysis indicated that *PGK1* may activate multiple immune genes and interfere with the tumor environment in 32 types of cancer (Figure 8D).

## 4 DISCUSSION

Tongue squamous cell carcinoma, characterized by a notably aggressive biologic behavior and heterogeneous survival, is a clinically, gnomically, and immunologically distinct subgroup of head and neck tumors that arise from the base of the tongue.^2^ Current treatments for TSCC, including surgical techniques and chemoradiotherapy combined with cetuximab have failed to achieve satisfactory therapeutic effects; thus, immune checkpoint inhibitors are expected to prolong OS in advanced TSCC.^22^ It is generally agreed that PD1 or PDL1 acted as major indicators to guide the selection of immune checkpoint inhibitors.^23^ However, there are conflicting opinions on these indicators in the literature. Sanni Tervo reported that the higher density and intensity of PD1 were significantly correlated with better survival.^24^ Yet Tomofumi Naruse and Naoki Akisada demonstrated that patients with high PD‐L1 expression had a remarkably shorter DFS and increased local recurrence.^25^, ^26^ Currently, PD1/PDL1 remains inapplicable in the prediction of TSCC prognosis; thus, it is urgent to elucidate the underlying genetic pathogenesis in TSCC and to identify reliable prognostic biomarkers based on the immune landscape.

In this study, we performed an ssGSEA of immune genes from the ImmPort database to calculate an immune‐based prognostic score and determine the specific immune infiltration, which is more generic than the normal gene signature. First, we conducted the ESTIMATE and CIBERSORT analyses to find the ssGSEA‐based clusters that were highly associated with ESTIMATE scores and immune scores. Furthermore, T cells, CD4 memory‐activated T cells, CD8 T cells, and follicular helper T cells were found to be remarkably increased in the cluster with a better prognosis. Many studies have reported that a higher degree of T cell infiltrates, especially CD8+, improved OS in HPV‐positive and HPV‐negative oral squamous carcinoma,^27^ and DFS in patients with respectable laryngeal cancer.^28^, ^29^ Moreover, some researchers found that the frequency of PD1+ CD8+ T cells in the tumor microenvironment, which negatively impacts effector and immunosuppressive functions, can predict the clinical efficacy of PD1 inhibitors better than PD1 or PD‐L1 expression or TMB.^30^ Next, we explored specific mRNAs that could potentially mediate CD8+ T cells and constructed a prognostic signature with a broad scope of applications that could accurately identify cases with worse survival. The results indicated that the constructed risk model was much better than the current TNM classification system, but its utility needs to be reverified with other TSCC groups.

In our analysis, we identified and selected three hub genes, *PGK1*, *GPI*, and *RPE*, which are involved in the glycolysis pathway. Glycolysis is the metabolism of glucose to lactate despite the presence of adequate oxygen, which is also called the Warburg effect.^31^ The Warburg effect has been recognized to be one of the hallmarks of cancer and to provide essential evidence that oncogenes and tumor suppressors alter the regulation of energy metabolism.^32^ More recent studies have found that glycolysis and lactate production, in particular, are strongly increased in peripheral T cells.^33^ Excessively increased glucose uptake induces T cell activation and proliferation and can lead to increased proliferation and lymphoproliferative disease.^34^ Our findings demonstrated a similar relationship between the tumor immune environment and glycolysis in TSCC.

Phosphoglycerate kinase 1 (*PGK1*), an isoform of the PGK family that catalyzes ATP formation in the glycolysis pathway,^35^ plays a rate‐limiting role in controlling ATP and 3‐PG levels^36^ and is a mediator in the regulation of autophagy initiation, DNA replication, and repair in mammal cell nuclei.^37^, ^38^, ^39^ More notably, via the pan‐cancer analysis in our study, the mRNA expression level of *PGK1* was significantly associated with the prognosis of multiple cancers, including head and neck, liver, and breast cancer. *PGK1* has gradually become a novel target in clinical research for various cancers. Researchers have found that enhanced glycolysis activity combined with higher *PGK1* expression in breast cancer was associated with pro‐tumor immunity via upregulation of immune/inflammation pathways, especially the IL‐17 signaling pathway.^40^ However, little is known about the relationship between PGK1 and the tumor immune microenvironment in TSCC. In this study, we first identified the key role of *PGK1* in TSCC and comprehensively evaluated its profile in the immune landscape. Additionally, we constructed an interactive network with *PGK1* at the core and many intersections were verified experimentally. Han RL reported that miR‐383 suppressed the expression level of *LDHA*, an mRNA highly correlated with *PGK1*, by directly binding to its 3'‐untranslated region and thus silenced aerobic glycolysis in ovarian cancer cells.^41^ Similarly, Fang found that overexpression of miR‐383 could inhibit cell proliferation and invasion triggered by LDHA in hepatocellular cancer.^42^ Furthermore, Li verified that circ_0136666 accelerated the progression of colorectal cancer by directly targeting and downregulating miR‐383.^43^ Hence, based on these reports, the interactive network constructed in this study was acceptable for the in‐depth exploration of in vitro experiments.

Glycosylphosphatidylinositol (*GPI*)‐anchored proteins have been regarded as well‐established cancer biomarkers that correlate with changes in GPI‐T expression. With GPI‐T, the importance of these proteins truly came to light only after their discovery in bladder cancer.^44^ Several interesting findings have demonstrated the highly intimate relationship between the levels of GPI‐T subunits and different cancer types.^45^ Ribulose‐phosphate 3‐epimerase (*RPE*), an important protein involved in carbohydrate degradation can catalyze the reversible epimerization of D‐ribulose 5‐phosphate to D‐xylulose 5‐phosphate.^46^ However, evidence supporting the oncogenic nature of *RPE*, particularly with respect to tumorigenesis, was minimal. Together, none of the past studies focused on the role of both biomarkers in TSCC; thus, our study is the first to unravel the genomic properties of these biomarkers in the field of immunology. Certainly, our findings in this study may be the tip of an iceberg, and there are some limitations that need to be improved; for instance, the mechanism underlying TSCC progression still has immense potential to be further elucidated, and further clinical trials and molecular experiments are required to verify our results.

## 5 CONCLUSION

This study systematically evaluated TSCC patients from the TCGA database and constructed different clusters based on ssGSEA analysis and profiled the immune landscape and immune cell infiltration in the tumor environment. After analyzing the correlation among clusters, we established a signature of 15 immune‐related genes that were identified as having independent prognostic significance for TSCC and selected three hub genes, *PGK1*, *GPI*, and *RPE*. Our findings suggest that these immune‐related genes may be promising indicators for the mediation of the immune microenvironment and may provide novel insights into immunotherapy for TSCC.

## CONFLICT OF INTEREST

The authors declare that they have no competing interest.

## AUTHOR CONTRIBUTION

KC designed the study. YJ analyzed, interpreted the data, wrote the original draft. ZWW, and DH wrote this manuscript. YXZ and YXC edited and revised the manuscript. All authors have seen and approved the final version of the manuscript.

## Supporting information

Fig S1

Table S1

Table S2

## Data Availability

The data that support the findings of this study are available from the corresponding author upon reasonable request.
