# Supplementary figures and images for: Identification of novel subtypes based on ssGSEA in immune‐related prognostic signature for tongue squamous cell carcinoma
Source: Cancer Med. 2021 Oct 20;10(23):8693–707. doi: 10.1002/cam4.4341 (PMC8633230; doi:10.1002/cam4.4341)

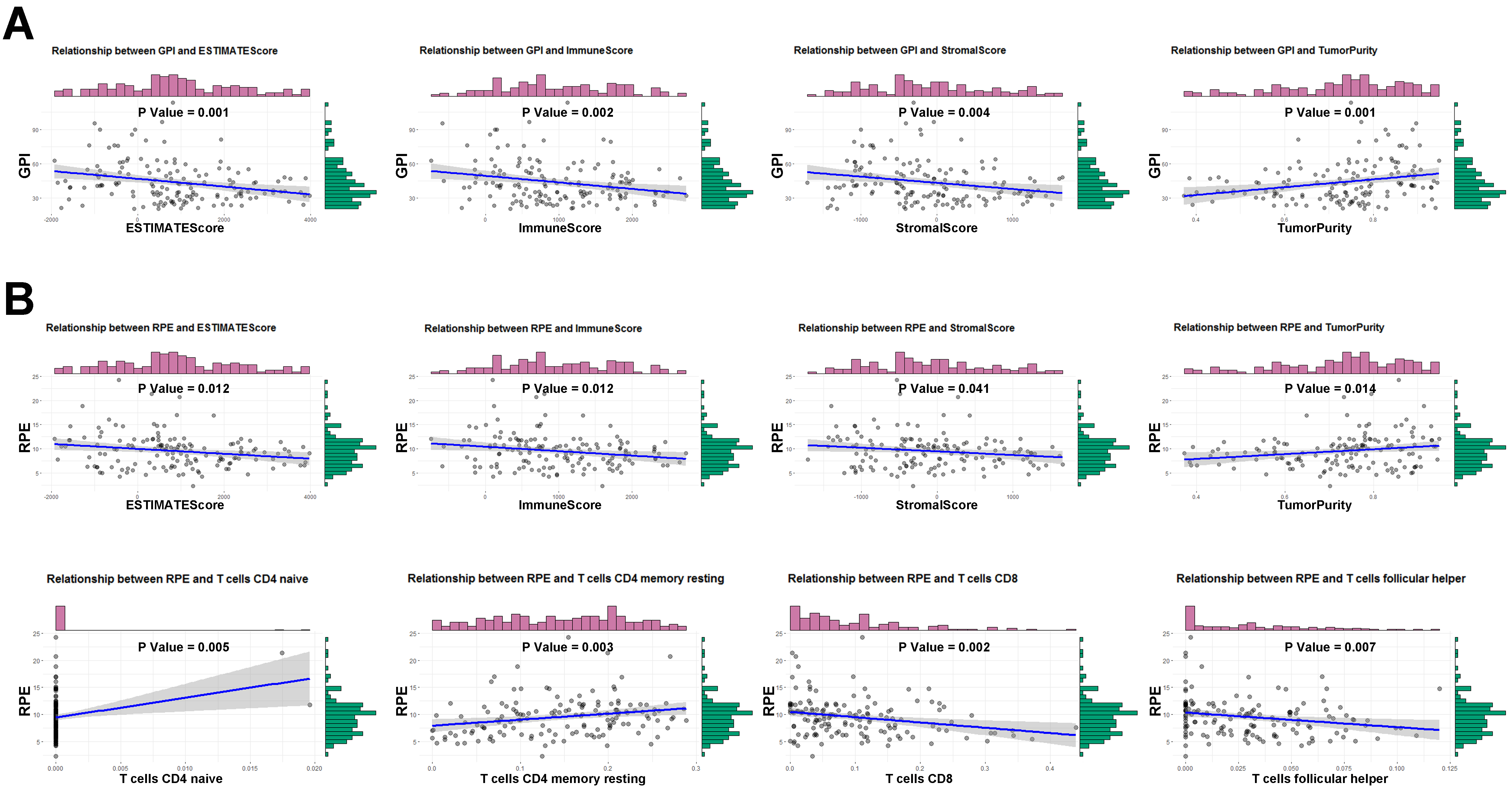

Supplement: Supplementary file 1 — Fig S1 [file CAM4-10-8693-s003.tif]
