# Supplementary material for: Identification of novel subtypes based on ssGSEA in immune‐related prognostic signature for tongue squamous cell carcinoma
Source: Cancer Med. 2021 Oct 20;10(23):8693–707. doi: 10.1002/cam4.4341 (PMC8633230; doi:10.1002/cam4.4341)
Supplement: Supplementary file 2 — Table S1 [file CAM4-10-8693-s001.docx]

**Supplementary Table S1.** The Cox analysis of 41 prognostic mediators.

| **ID** | **HR** | **HR.95L** | **HR.95H** | ***p*-Value** |
| --- | --- | --- | --- | --- |
| *UTP4* | 1.066063 | 1.009302 | 1.126016 | 0.021927 |
| *SNAPC1* | 1.042653 | 1.008886 | 1.077549 | 0.012895 |
| *AREG* | 1.003422 | 1.000161 | 1.006693 | 0.039682 |
| *CCDC43* | 1.158766 | 1.033649 | 1.299028 | 0.011482 |
| *CXCL8* | 1.001685 | 1.00041 | 1.002961 | 0.009563 |
| *CCL22* | 0.893369 | 0.818861 | 0.974656 | 0.011158 |
| *STK17A* | 1.013405 | 1.001917 | 1.025025 | 0.022066 |
| *KLHL2* | 1.095186 | 1.019119 | 1.176931 | 0.013301 |
| *PTP4A1* | 1.023185 | 1.008398 | 1.03819 | 0.00203 |
| *SH2D3C* | 0.782621 | 0.633128 | 0.967411 | 0.023432 |
| *ENO1* | 1.001296 | 1.000163 | 1.002431 | 0.025007 |
| *ANKRD22* | 0.963427 | 0.932332 | 0.995558 | 0.02602 |
| *PRPF4* | 1.045317 | 1.00465 | 1.08763 | 0.02859 |
| *SUN1* | 1.068127 | 1.018115 | 1.120596 | 0.007066 |
| *TPSAB1* | 0.93748 | 0.885105 | 0.992953 | 0.027732 |
| *IARS* | 1.069399 | 1.024848 | 1.115886 | 0.001998 |
| *CD5* | 0.858476 | 0.740253 | 0.99558 | 0.043532 |
| *NTMT1* | 1.072035 | 1.014507 | 1.132826 | 0.013446 |
| *IER3* | 1.00489 | 1.000604 | 1.009195 | 0.025298 |
| *FAM210A* | 1.12729 | 1.044234 | 1.216951 | 0.002152 |
| *CCR7* | 0.867596 | 0.766334 | 0.982238 | 0.024898 |
| *ANLN* | 1.025136 | 1.001242 | 1.0496 | 0.039098 |
| *FAT1* | 1.019781 | 1.00128 | 1.038625 | 0.036002 |
| *PGK1* | 1.00557 | 1.00232 | 1.00883 | 0.00077 |
| *TMEM106B* | 1.082584 | 1.000025 | 1.171958 | 0.049927 |
| *IPO5* | 1.052444 | 1.010625 | 1.095995 | 0.01348 |
| *SFTA1P* | 1.041754 | 1.001862 | 1.083233 | 0.040037 |
| *ISCA1* | 1.09345 | 1.012731 | 1.180602 | 0.022414 |
| *SRP72* | 1.036316 | 1.007717 | 1.065726 | 0.012478 |
| *CTSG* | 0.752432 | 0.583751 | 0.969855 | 0.02807 |
| *PFKP* | 1.025862 | 1.007326 | 1.044739 | 0.006059 |
| *ABCE1* | 1.052708 | 1.00885 | 1.098472 | 0.017992 |
| *DARS* | 1.043543 | 1.011706 | 1.076382 | 0.007014 |
| *ADM* | 1.0068 | 1.000117 | 1.013527 | 0.046109 |
| *GPI* | 1.014747 | 1.000517 | 1.02918 | 0.042188 |
| *PDGFA* | 1.060698 | 1.009132 | 1.114899 | 0.020477 |
| *SECTM1* | 1.018818 | 1.001309 | 1.036633 | 0.035042 |
| *LTB* | 0.943895 | 0.893199 | 0.997468 | 0.040366 |
| *CCR4* | 0.681466 | 0.483982 | 0.959531 | 0.028051 |
| *MTHFD1L* | 1.078766 | 1.017053 | 1.144222 | 0.01165 |
| *RPE* | 1.07985 | 1.011023 | 1.153363 | 0.022243 |
