# Supplementary material for: Identification of novel subtypes based on ssGSEA in immune‐related prognostic signature for tongue squamous cell carcinoma
Source: Cancer Med. 2021 Oct 20;10(23):8693–707. doi: 10.1002/cam4.4341 (PMC8633230; doi:10.1002/cam4.4341)
Supplement: Supplementary file 3 — Table S2 [file CAM4-10-8693-s002.docx]

**Supplementary Table S2.** The degree of hub genes calculated by the Cytoscape (v3.6.1) plugin cytoHubba.

| **node_name** | **MCC** | **DMNC** | **MNC** | **Degree** | **EPC** | **BottleNeck** | **EcCentricity** | **Closeness** | **Radiality** | **Betweenness** | **Stress** | **Clustering Coefficient** |
| --- | --- | --- | --- | --- | --- | --- | --- | --- | --- | --- | --- | --- |
| *PGK1* | 4 | 0.30898 | 3 | 3 | 3.344 | 2 | 0.14583 | 4.08333 | 2.91667 | 4 | 8 | 0.66667 |
| *GPI* | 4 | 0.30898 | 3 | 3 | 3.332 | 1 | 0.14583 | 4.08333 | 2.91667 | 4 | 8 | 0.66667 |
| *RPE* | 3 | 0.30779 | 2 | 3 | 3.42 | 7 | 0.19444 | 4.33333 | 3.11111 | 18 | 24 | 0.33333 |
| *KLHL2* | 3 | 0 | 1 | 3 | 2.651 | 5 | 0.20833 | 3.5 | 1.5625 | 10 | 10 | 0 |
| *IER3* | 2 | 0 | 1 | 2 | 2.413 | 2 | 0.20833 | 3 | 1.45833 | 6 | 6 | 0 |
| *IARS* | 2 | 0.30779 | 2 | 2 | 2.985 | 1 | 0.11667 | 3.28333 | 2.52778 | 0 | 0 | 1 |
| *CCR7* | 2 | 0 | 1 | 2 | 2.966 | 7 | 0.19444 | 3.83333 | 3.01389 | 16 | 20 | 0 |
| *CCL22* | 2 | 0 | 1 | 2 | 2.584 | 2 | 0.14583 | 3.41667 | 2.72222 | 10 | 12 | 0 |
| *NTMT1* | 1 | 0 | 1 | 1 | 2.084 | 1 | 0.13889 | 2.33333 | 1.25 | 0 | 0 | 0 |
| *SUN1* | 1 | 0 | 1 | 1 | 1.961 | 1 | 0.13889 | 2.16667 | 1.14583 | 0 | 0 | 0 |
| *CTSG* | 1 | 0 | 1 | 1 | 2.001 | 1 | 0.11667 | 2.53333 | 2.23611 | 0 | 0 | 0 |
| *CCDC43* | 1 | 0 | 1 | 1 | 1.999 | 1 | 0.13889 | 2.33333 | 1.25 | 0 | 0 | 0 |
